# Effects of thinning and understory removal on the soil water-holding capacity in *Pinus massoniana* plantations

**DOI:** 10.1038/s41598-021-92423-5

**Published:** 2021-06-22

**Authors:** Ting Wang, Qing Xu, Deqiang Gao, Beibei Zhang, Haijun Zuo, Jing Jiang

**Affiliations:** 1 Key Laboratory of Forest Ecology and Environment of National Forestry and Grassland Administration, Research Institute of Forest Ecology, Environment and Protection, Chinese Academy of Forestry, Beijing, 100091 China; 2 University of Calgary, Calgary, T2N1N4 Canada

**Keywords:** Forest ecology, Forestry

## Abstract

Forest management practices play an important role in regulating the soil water-holding capacity of plantation. However, most studies focus on soil water dynamics present during large-scale forest loss and afforestation events, while little is known about how soil water under different forest management practices responds to rainfall events and which factors mainly regulate soil water-holding capacity. In this study, a stable hydrogen isotope was used to explore the contribution of three natural rainfall events (8.9, 13.3 and 67.7 mm) to soil water (CRSW) in a *Pinus massoniana* plantation under four forest management practices (no thinning (NTN), understory removal (USR), light-intensity thinning (LIT) and heavy-intensity thinning (HIT)) in the Three Gorges Reservoir Area of the Yangtze River Basin in China. Furthermore, a structural equation model was employed to determine the effects of vegetation biomass and soil properties on the CRSW. The results showed that plantation soil under different forest management practices exhibited different water-holding capacities. Following light (8.9 mm) and moderate (13.3 mm) rainfall events, the CRSW in the HIT stand was slightly higher than that in the other stands. Following heavy (66.7 mm) rainfall event, the CRSW of most layers in USR stand was not different from the other three stands, while the CRSW in the LIT and NTN stands was significantly higher than that in the HIT stand in the 0–100 cm soil layers, suggesting that soil in the LIT and NTN stands had a greater water-holding capacity than that in the HIT stand. In addition, soil properties were the main factors directly affecting the CRSW, explaining 60% and 37% of the variation in the CRSW on the first and seventh days after heavy rainfall, respectively. Overall, compared to the HIT stand, the LIT and NTN stands showed greater capacity in retaining rainwater. Therefore, under expected global changes with frequent occurrences of extreme precipitation events, methods involving light-intensity and no thinning should be employed to build up soil and water conservation functions, which will be critical for keeping water-holding capacity and moderating floods.

## Introduction

Soil water, as the main component of the hydrologic cycle in plantation ecosystems, plays an important role in mass and energy transformation^1^. The spatial and temporal distribution of soil water is affected by various factors such as precipitation, vegetation cover, and soil properties^2–4^. Among them, the amount of precipitation directly influences soil water content and dynamics and determines the growth, composition, structure, and function of vegetation in plantations^5–8^. However, under anticipated climate change processes, plantations will be susceptible to changes in precipitation patterns due to their monospecific structures and poor nutrition conditions^9–11^. Forest management practices, by optimizing vegetation structures and functions and improving soil water-holding capacity, can increase forest productivity, delay runoff generation, slow soil erosion and reduce flooding^12–14^. Therefore, it is essential to understand soil water responses to different magnitudes of rainfall in plantations under different forest management practices.

Thinning and understory removal are commonly adopted forest management practices^15–17^. Reasonable thinning intensity and proper understory vegetation management reduce water and nutrient competition between tree species, which enhances forest resistance and resilience to global changes and human disturbances^16,18,19^. Meanwhile, thinning and understory removal have a considerable influence on the spatial and temporal distribution of soil water and soil water-holding capacity in forest ecosystems^16,17^. These effects vary greatly across different site conditions, plant species, and thinning intensities^18^. However, it is unclear whether thinning and understory removal have positive impacts on soil water-holding capacity. For instance, Wang et al.^13^ found that thinning reduced precipitation interception and plant water use and further decreased soil water consumption. In contrast, some studies have indicated that following thinning, soil water evaporation and forest and understory transpiration increase, possibly offsetting or reversing the effects of thinning on forest water consumption^14,17,20^. Overall, the effects of thinning and understory removal on soil water distribution needs to be explored further.

Soil water, as a result of long-term biophysical processes, is determined by multiple factors^21–24^. Previous studies have indicated that soil water patterns were regulated by both vegetation^25,26^ and soil properties^27–29^. However, little is known about which factors drive soil water distribution under different forest management practices (i.e., thinning and understory removal). On the one hand, thinning and understory removal affected forest hydrological processes such as precipitation interception and vegetation evapotranspiration by regulating vegetation structures (stand density, the leaf area index, and canopy storage) and ultimately influenced soil water dynamics^30,31^. On the other hand, thinning and understory removal also impacted soil properties potentially attributable to the vegetation regulation and soil compaction^32,33^. Forest management practices can cause soil compaction that leads to increased soil bulk density, decreased soil porosity, decreased permeability, decreased water holding capacity, decreased soil productivity and a decreased infiltration rate^12,32,34^. These effects can, in turn, increase the potential for overland flow and can accelerate soil erosion^29^. Hence, it is necessary to explore the main factors that determine soil water-holding capacity under different forest management practices.

*Pinus massoniana* Lamb. is a common, widely distributed forest species with strong adaptability and tolerance to harsh environments in subtropical areas of China^35^. As an ecological construction and timber tree species, *P. massoniana* plays a crucial role in restoring ecological integrity and providing forest resources in the Three Gorges Reservoir Area of China^36^. Due to human disturbances and poor management, the productivity of *P. massoniana* plantations in the region is low^37^. In recent years, the structure reconstruction of *P. massoniana* plantations have been implemented in the area through the adoption of forest management practices. However, the effects of different forest management practices on soil water dynamics and the soil capacity of retaining rainfall in *P. massoniana* plantations have not been well examined. In this study, we employed stable hydrogen isotope to examine contribution of rainfall to soil water in *P. massoniana* plantations under different forest management practices. We further examined vegetation and soil properties to identify the factor that was the best predictor in regulating contribution of rainfall to soil water in the studied *P. massoniana* plantation. We addressed the following two questions. (1) How do different forest management practices (thinning and understory removal) affect the contribution of rainfall to soil water? (2) Is vegetation or soil the main factor that influences soil water-holding capacity?

## Materials and methods

### Study area and sampling design

**Figure 1:**
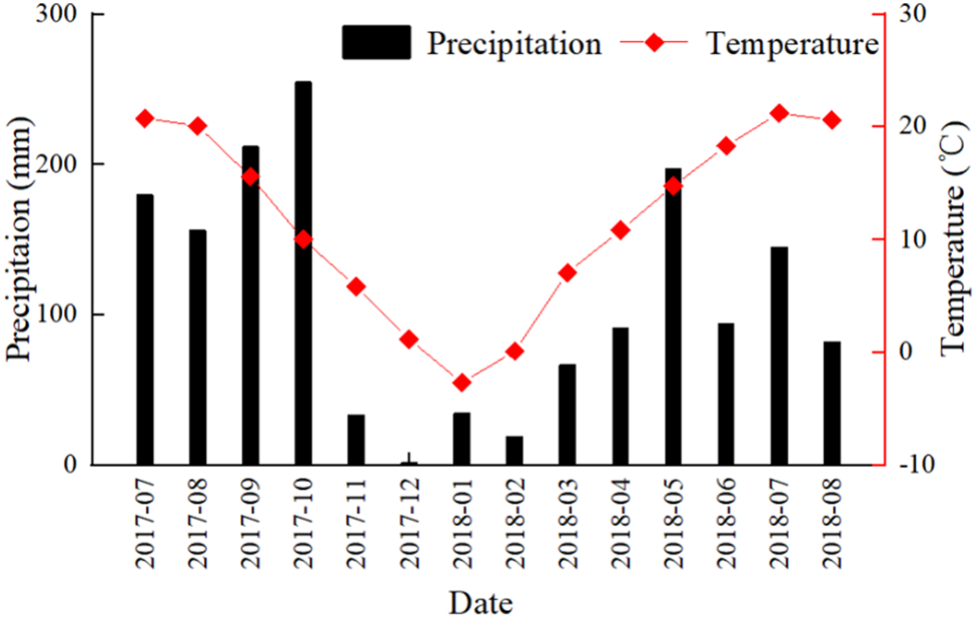
Monthly mean air temperature (line) and precipitation (bar) from July 2017 to August 2018 in the Three Gorges Reservoir Area.

The study area (30°59′ N, 110°47′ E) is located in the Jiulingtou Region of the Three Gorges Reservoir Area of the Yangtze River Basin (Supplementary Fig. S1). The area belongs to a subtropical monsoon climate zone with a mean annual temperature of 16.9 °C and mean annual precipitation levels of 1000–1250 mm. The precipitation mainly occurs from May to October (Fig. 1). The Three Gorges Reservoir Area is prone to rainstorms with 9–15 heavy rainfall events of daily accumulative precipitation levels of ≥ 25 mm occurring per year. Heavy precipitation often leads to soil erosion and natural disasters such as collapse, landslides and debris flows. Soil in the area is mainly composed of loamy yellow brown soil. The soil particle composition in surface soil layer (0–10 cm) in the *P. massoniana* stands are 29.01 ± 2.18%, 29.21 ± 10.36% and 41.78 ± 11.93% for sand, silt and clay, respectively^38^. The soil properties are shown in the Supplementary Tables S1 and S2.

The *P. massoniana* plantation was established through aerial seeding in the 1970s. *P. massoniana* is the main species present and is evenly distributed across the study area. Accompanying tree species include *Betula luminifera*, *Cunninghamia lanceolata* and *Toxicodendron vernicifluum*. Undergrowth shrubs mainly include *Litsea cubeba*, *Lespedeza bicolour* and *Pyracantha fortuneana,* and understory herbaceous plants include *Woodwardia japonica*, *Carex tristachya* and *Aster ageratoides*. At the beginning of the present experiment, the forests had a stocking of 1700 stems per ha. In the 43-year-old *P. massoniana* forest of the Jiulingtou Region, four different forest management practices (no thinning (NTN, without human disturbance), understory removal (USR, all understory vegetables removed), light-intensity thinning (LIT, 15% basal area of canopy trees removed) and heavy-intensity thinning (HIT, 70% basal area of canopy trees removed)) were applied with chainsaw carried by manpower in September 2013. Among them, the USR stands were removed understory once a year. All harvested vegetation and residues were removed from the treatment areas. All management practices were cutting at ground level and did not deal with surface litter and underground root systems. Each treatment repeated three times (n = 3 plantations). Twelve (4 treatments × 3 replications per treatment) 20 m × 20 m plots laid out in similar site conditions and separated from each other by at least 20 m. In order to avoid soil compaction resulting from heavy machinery and vehicles, all logging and harvesting operations were completed by manpower. The characters of plantations obtained in 2017 are summarized in Table 1.

**Table 1:** Characteristics of the *P. massoniana* plantation with no thinning (NTN), understory removal (USR), light-intensity thinning (LIT) and heavy-intensity thinning (HIT) treatments in 2017.

Treatment	Geographic location	Altitude (m)	Slope grade (°)	Exposure	Canopy density	DBH (cm)
NTN	30°59′ 20″ N 110°47′ 08″ E	1225	34	NW	0.73	12.06
USR	30°59′ 23″ N 110°47′ 07″ E	1240	35	NW	0.70	13.60
LIT	30°59′ 21″ N 110°47′ 05″ E	1220	33	NW	0.60	18.86
HIT	30°59′ 23″ N 110°47′ 09″ E	1226	33	NW	0.30	10.40

### Sampling collection

According to meteorological standards, three different magnitudes of rainfall events were defined as 24-h amount of rainfall, including light (0–10 mm), moderate (10–25 mm) and heavy (> 25 mm) rainfall events. Samples collected the day before a rainfall event were taken as pre-rainfall control samples for comparison to post-rainfall samples. The light rainfall event involved 8.9 mm of precipitation occurring on March 20, 2018 with the samples collected from March 21 to 29. The moderate rainfall event involved 13.3 mm of precipitation occurring on August 17, 2018 with the samples collected from August 19 to 27. The heavy rainfall event involved 67.7 mm of precipitation occurring from July 17 to 19, 2017 with the samples collected from July 20 to 26. The characteristics of rainfall events are shown in the Supplementary Fig. S2.

Rainwater samples were collected after each of the three rainfall events with three rain gauges on an open site near the studied forest. A table tennis ball was placed in the funnel of each gauge to limit evaporation^39^. At the end of each rainfall event, the amount of precipitation was recorded. Rainwater samples collected from the three rain gauges were mixed in a sampling bottle. Soil water samples were collected from five layers (0–20, 20–40, 40–60, 60–80, and 80–100 cm) with soil auger every other day until the next rainfall. Two subsamples were collected from each of the soil samples: one was stored in a freezer for isotopic analysis, and another was used to obtain the soil water content (SWC, %) as determined by drying at 105 °C for 24 h. All collected samples were immediately placed into sampling bottles, sealed with Parafilm, stored in a portable cooler (− 5 to 0℃) in the field, and then transported to the laboratory. All of the samples were stored in a refrigerator at − 16 °C in the laboratory until isotope analysis^40^.

### Isotopic analyses

Soil water was extracted by cryogenic vacuum distillation^41^. The stable hydrogen isotope ratios of rainwater and soil water samples were analysed using an isotope ratio mass spectrometer (Delta V Advantage, Thermo Fisher Scientific, Inc., Waltham, Massachusetts, USA) coupled with an elemental analyser (Flash 2000 HT, Thermo Fisher Scientific, Inc.) in the joint stable isotope laboratory between Shenzhen Huake Precision Analytical, Inc. and Tsinghua Shenzhen International Graduate School. The precision of *δ*D was ± 1‰ based on three internal standards after calibration with Vienna Standard Mean Ocean Water standards (V-SMOW)^42^. The hydrogen isotopic ratio was expressed as follows (Eq. 1):

1 $$\delta {\text{D}} = {}[\left( {{\text{R}}_{{{\text{sample}}}} /{\text{R}}_{{{\text{standard}}}} } \right)1] \times 1000\permil$$

where R_sample_ and R_standard_ are the D/H molar ratio of the sample and the V-SMOW standards, respectively.

### Contribution of rainfall to soil water (CRSW)

The source of soil water was determined by comparing the soil water *δ*D with the potential water source *δ*D. The soil water came from two sources: rainwater and pre-event water^43^. Therefore, a two-end linear mixing model we used to determine the ratio of the two water sources^4^. The relative contribution of rainfall to soil water (CRSW) was calculated with the following equations.

2 $$\delta {\text{D}}_{{{\text{SW}}}} = {\text{ }}f_{{\text{R}}} \times \delta {\text{D}}_{{\text{R}}} + {\text{ }}f_{{{\text{PW}}}} \times \delta {\text{D}}_{{{\text{PW}}}}$$

3 $$f_{{\text{R}}} + {\text{ }}f_{{{\text{PW}}}} = {\text{ 1}}$$

4 $$f_{{\text{R}}} = {\text{ }}(\delta {\text{D}}_{{{\text{SW}}}} - \delta {\text{D}}_{{{\text{PW}}}} )/(\delta {\text{D}}_{{\text{R}}} - \delta {\text{D}}_{{{\text{PW}}}} )$$

5 $${\text{CRSW }} = {\text{ }}f_{{\text{R}}} \times {\text{ 1}}00\%$$

where ƒ_R_ and ƒ_PW_ are the proportions of soil water obtained from rainwater and pre-event water, respectively. *δ*D_R_, *δ*D_SW_, and *δ*D_PW_ denote the deuterium isotope ratios of rainwater, soil water, and pre-event water, respectively. Equation (4) was solved from Eqs. (2) and (3). Equation (5) was used to calculate the CRSW.

### Determination of vegetation biomass and soil properties

To determine which factors might affect the CRSW, vegetation biomass (tree, litter and root biomass) was measured. Tree biomass was estimated by measuring the height and diameter at the breast height (DBH) of each tree in each plot, and these parameters were incorporated into the allometric growth model for *P. massoniana, C. lanceolata* and broadleaf tree species to obtain the aboveground biomass for each tree^44^. The biomass for all trees in each plot was summed as the total tree biomass. For litter, three 1 × 1 m quadrats were established to collect all litter above ground in each plot. The root biomass in 1 × 1 m quadrats was divided into five layers corresponding to the soil samples. All biomass samples were weighed after being dried in an oven to constant weight.

We also measured a suite of soil properties to explore the effects of edaphic variables on the CRSW. For each soil depth, a fixed volume of 100 cm^3^ ring cutter with undisturbed soil was obtained. The soil samples were then oven dried, and bulk density was calculated from the ratio of dry soil mass to the volume of ring cutter. Soil total porosity and field capacity were measured according to the cutting ring method^45^. The ring cutter with undisturbed soil was soaked in water for 12 h to fully saturate, and then placed on coarse sand for 2 h, 12 h and 24 h to drain the excess water from the soil pores, and calculated the noncapillary porosity, capillary porosity, total porosity and field capacity. We also established four control plots beside the four studied stands and analysed their soil properties, which to detect the differences in soil properties among the four studied stands can be attributed to forest management practices or to primitive soil differences. All methods were guided on “Observation Methodology for Long-term Forest Ecosystem Research” of National Standards of the People's Republic of China (GB/T 33027-2016).

### Data analysis

Levene's test was used to test the homogeneity of variance. An independent t-test was performed to compare soil water *δ*D of the pre- and post-rainfall. A one-way ANOVA was conducted to measure the effect of different forest management practices on the CRSW, soil properties, and vegetation biomass. Multiple comparisons were used to further determine differences between the different forest management practices. Statistical test results were considered significant at *P* < 0.05. An ordinary least squares regression was performed to evaluate the relationships between the CRSW and soil properties and vegetation biomass across the four treatments.

Structural equation modelling (SEM) was used to test the relative contributions of soil properties, tree biomass, litter and root biomass to the CRSW (AMOS 21.0 software, Amos Development Corporation, Chicago, IL, USA) (Supplementary Fig. S3). Given the close correlation found between the soil property variables, a principal component analysis (PCA) was used to obtain a comprehensive index representing soil properties before the SEM analysis. Variables that were significantly correlated with the CRSW were added to the dataset for the PCA. The first principal component (PC1) explained 93% of the total variance and was used as a new variable for SEM analysis (Supplementary Table S3). The model fit was assessed from chi-square tests (*χ*^*2*^) and the root mean square error of approximation (RMSEA)^46^.

## Results

### Temporal variations in soil water content following different rainfall events

**Figure 2:**
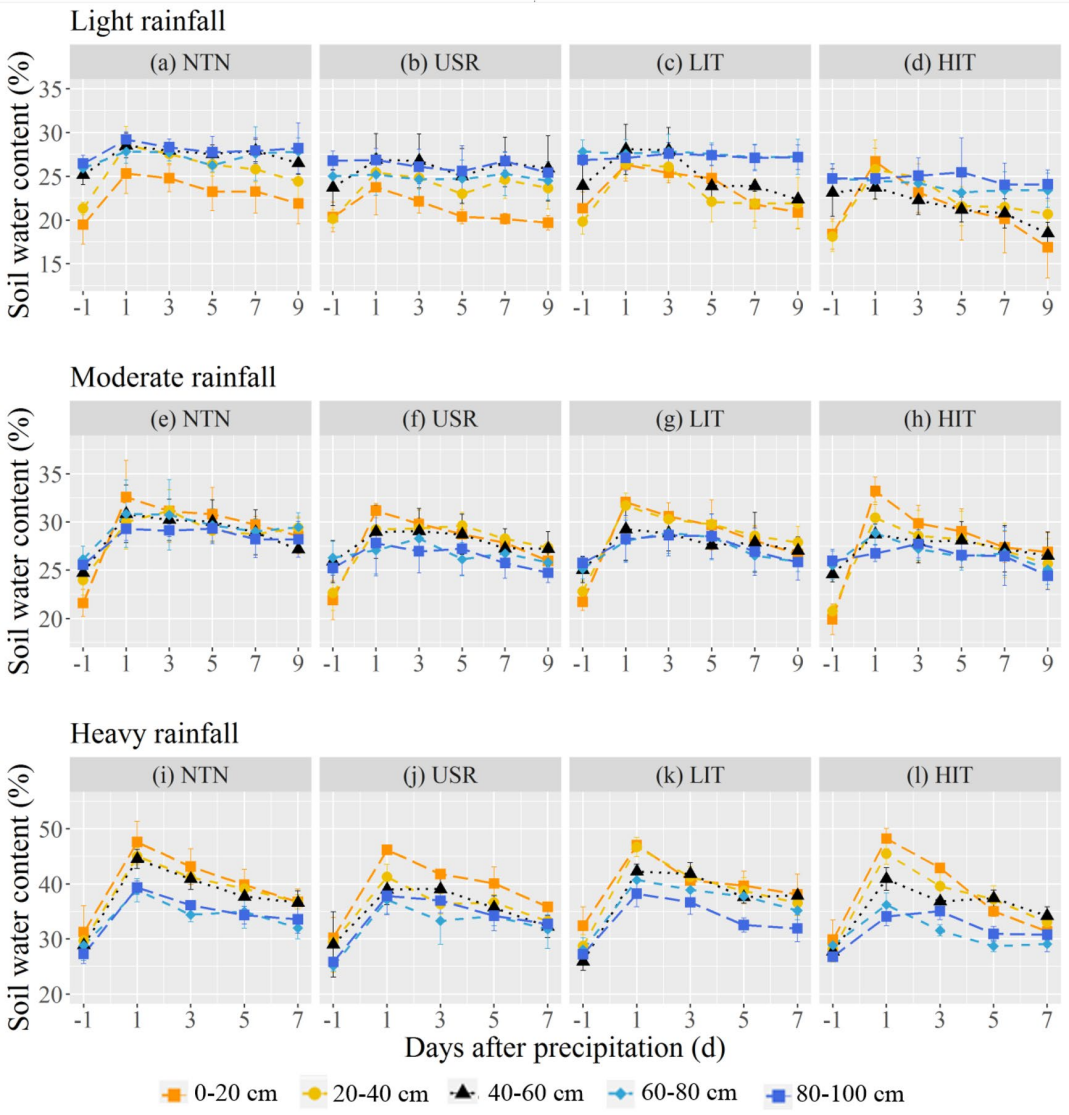
Daily soil water content (SWC) (mean ± SD, n = 3) of the *P. massoniana* stands with no thinning (NTN, **a,e,i**), understory removal (USR, **b,f,j**), light-intensity thinning (LIT, **c,g,k**) and heavy-intensity thinning (HIT, **d,h,l**) after the three rainfall events.

The SWC in the four stands varied with rainfall events and soil depth (Fig. 2). After an 8.9 mm rainfall event, the SWC increased with soil depth. The SWC in the 0–60 cm soil layers of the four stands gradually decreased over 9 days following rainfall, while the SWC in the 60–100 cm soil layers remained stable (Fig. 2a–d). However, after 13.3 mm and 67.7 mm of rainfall, the SWC decreased with soil depth. The SWC in all five soil layers of the four stands decreased gradually over 7–9 days after rainfall (Fig. 2e–l). The SWC of the shallow soil layers (0–20 cm) was highly variable compared with levels of the other layers over the sampling periods (Fig. 2).

### Temporal variations in soil water δD following different rainfall events

**Figure 3:**
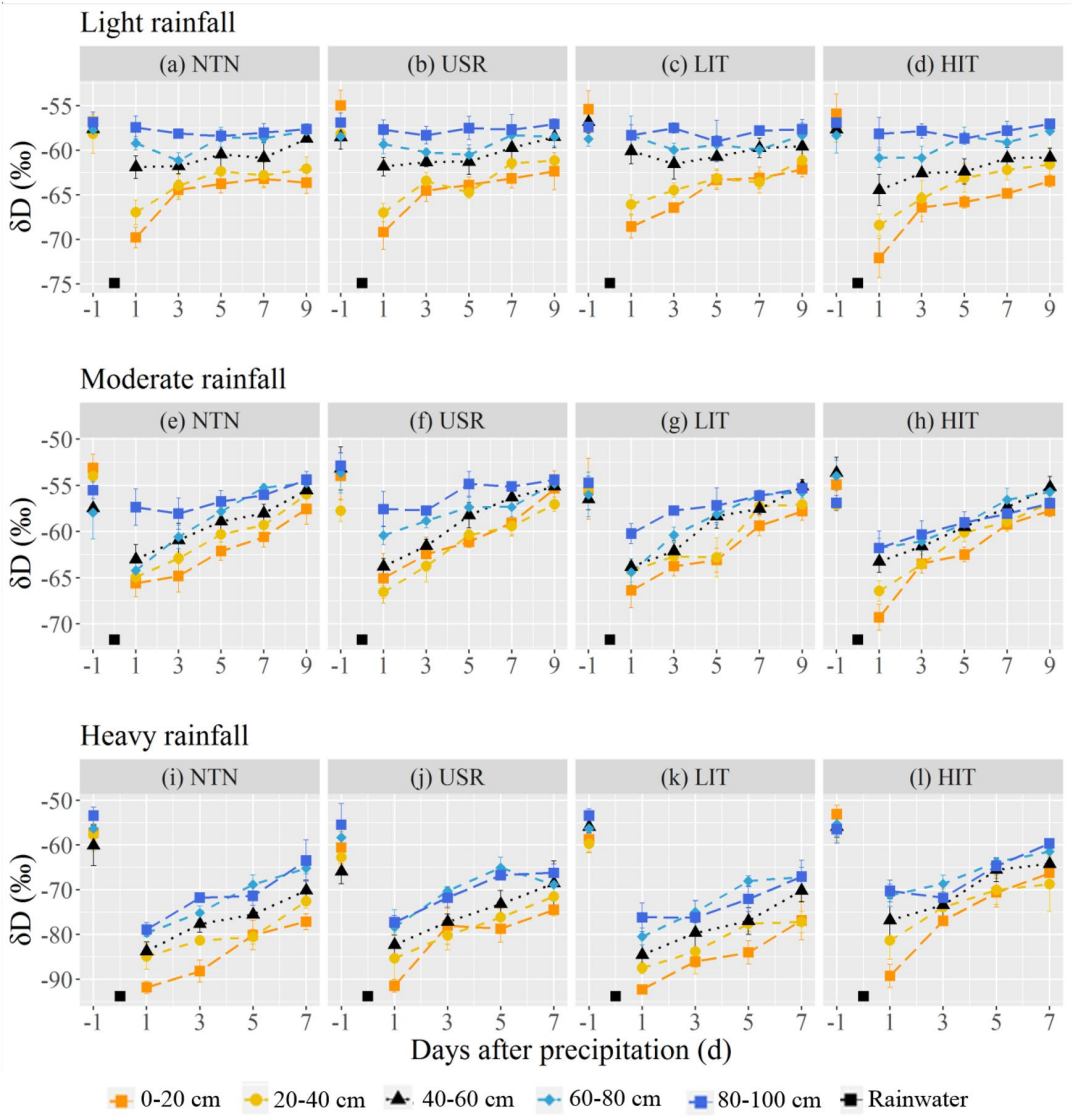
Daily *δ*D of rainwater and soil water of the *P. massoniana* stands with no thinning (NTN, **a,e,i**), understory removal (USR, **b,f,j**), light-intensity thinning (LIT, **c,g,k**) and heavy-intensity thinning (HIT, **d,h,l**) before and after the three rainfall events.

Over 7 or 9 days after the three rainfall events, soil water *δ*D varied between the rainwater *δ*D and pre-event water *δ*D, suggesting that soil water in these four *P. massoniana* stands mainly originated from rainwater and pre-event water. After a light rainfall event, the soil water *δ*D in the 0–40 cm soil layers of the NTN, USR, and LIT stands (Fig. 3a–c; Supplementary Table S4) and at 0–60 cm in the HIT stand (Fig. 3d; Supplementary Table S4) significantly decreased the first day after rainfall while the soil water *δ*D in deeper layers remained stable, indicating that light rainfall could infiltrate the 0–40 cm soil layers of the NTN, USR, and LIT stands and the 0–60 cm soil layers of the HIT stand. After a moderate rainfall event, the soil water *δ*D of the 0–80 cm soil layers of the four stands decreased on the first day (Fig. 3e–h; Supplementary Table S4) and then gradually increased over 9 days after rainfall, indicating that rainwater could infiltrate the 80 cm soil layer. Similarly, after heavy rainfall, the soil water *δ*D of the four stands decreased significantly on the first day (Fig. 3i–l; Supplementary Table S4) and then gradually increased over 7 days after the rainfall.

The coefficient of variation (CV) of the SWC and soil water *δ*D was the highest after heavy rainfall and the lowest after light rainfall (Table 2), indicating that soil water responses to rainfall depended on rainfall levels. The change in the CV of the SWC and soil water *δ*D among the different forest management practices showed that the SWC and soil water *δ*D of the NTN and LIT stands were relatively stable after rainfall while the most unstable values were in the HIT stand (Table 2).

**Table 2:** Summary statistics of soil water content (SWC) and soil water *δ*D after the three different rainfall events in the no thinning (NTN), understory removal (USR), light-intensity thinning (LIT) and heavy-intensity thinning (HIT) stands.

Treatment	Light rainfall				Moderate rainfall				Heavy rainfall			
	*δ*D		SWC		*δ*D		SWC		*δ*D		SWC	
	Mean ± SD (‰)	CV (%)	Mean ± SD (%)	CV (%)	Mean ± SD (‰)	CV (%)	Mean ± SD (%)	CV (%)	Mean ± SD (‰)	CV (%)	Mean ± SD (%)	CV (%)
NTN	−61.47 ± 3.51	5.71	26.72 ± 2.55	9.55	−59.80 ± 3.55	5.99	29.70 ± 2.49	8.38	−76.71 ± 7.33	9.56	38.64 ± 4.59	11.88
USR	−61.43 ± 3.50	5.70	24.55 ± 3.05	12.44	−59.41 ± 3.64	6.62	27.81 ± 2.34	8.43	−75.04 ± 7.36	9.81	36.77 ± 4.29	11.65
LIT	−61.25 ± 3.33	5.44	25.50 ± 2.98	11.70	−60.12 ± 3.57	5.93	28.48 ± 2.47	8.67	−77.74 ± 7.37	9.49	39.02 ± 4.23	10.83
HIT	−62.31 ± 4.00	6.43	22.85 ± 3.22	14.09	−60.81 ± 3.74	6.15	27.65 ± 2.68	9.70	−70.48 ± 7.60	10.79	36.26 ± 5.27	14.53

### Contribution of rainfall to soil water

**Figure 4:**
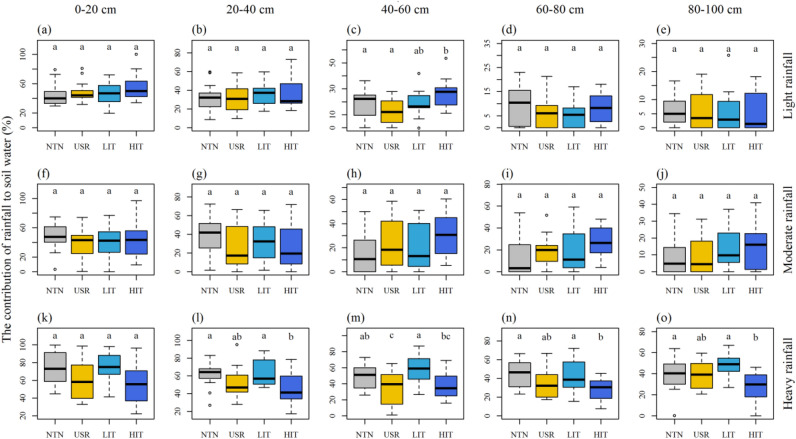
Contribution of light **(a–e)**, moderate **(f–j)**, and heavy **(k–o)** rainfall to soil water in five layers (0–20, 20–40, 40–60, 60–80, and 80–100 cm) with no thinning (NTN), understory removal (USR), light-intensity thinning (LIT) and heavy-intensity thinning (HIT) treatments. Different letters indicate significant differences at *P* < 0.05.

Over 9 days after light rainfall, the CRSW of the 0–40 cm soil layers (Fig. 4a,b; Supplementary Table S5) and 60–100 cm soil layers did not significantly differ across the four stands (Fig. 4d,e; Supplementary Table S5) while the CRSW of the 40–60 cm layer in the HIT stand was higher than those in the NTN and USR stands (Fig. 4c; Supplementary Table S5). Similarly, over 9 days after moderate rainfall, no significant difference in the CRSW was found in the 0–100 cm soil layers of the four stands (Fig. 4f–j; Supplementary Table S5). However, over 7 days after heavy rainfall, the CRSW in each layer differed among these stands. The CRSW was significantly lower in the HIT stand than in the NTN and LIT stands (Fig. 4k–o; Supplementary Table S5). No significant differences in the CRSW were found among the NTN, LIT and USR stands (Fig. 4k–o; Supplementary Table S5) in most soil layers over 7 days after heavy rainfall.

### Soil and vegetation factors affecting the CRSW after heavy rainfall

**Figure 5:**
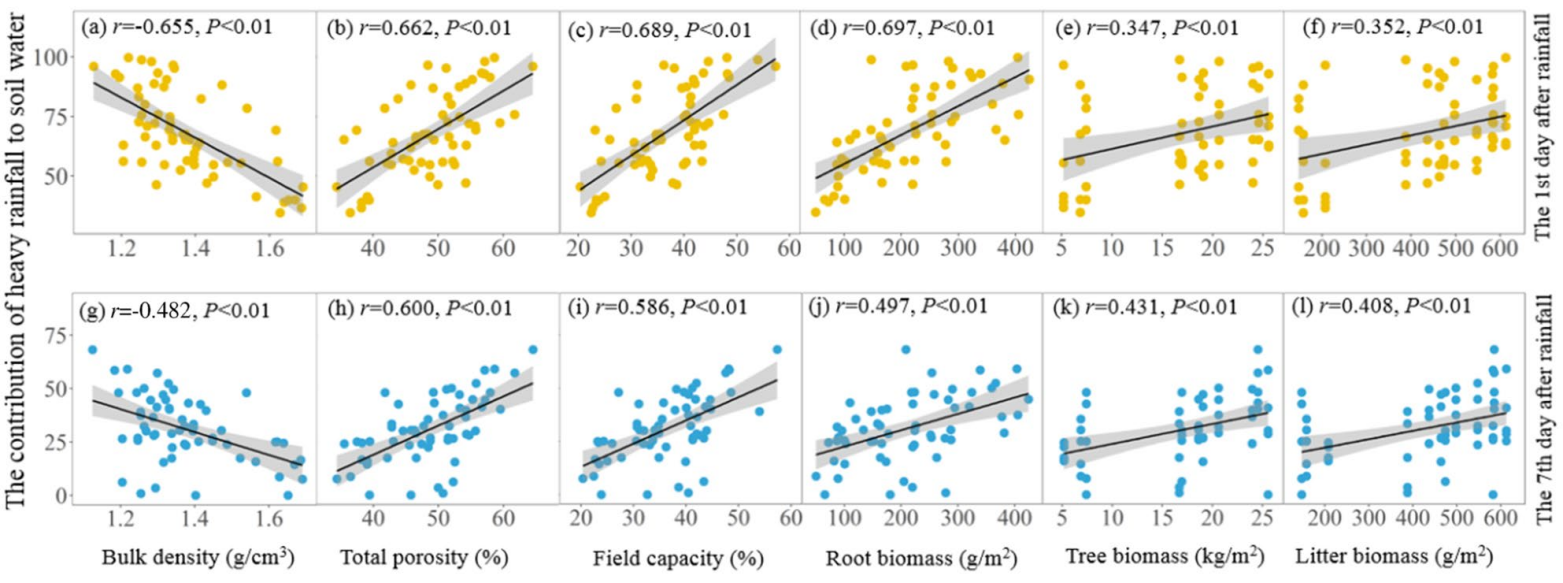
Relationships between the contributions of heavy rainfall to soil water and bulk density **(a,g)**, total porosity **(b,h)**, field capacity **(c,i)**, root biomass **(d,j)**, tree biomass **(e,k)** and litter biomass **(f,l)** on the first and seventh days after heavy rainfall.

The CRSW of the four stands was significantly correlated with soil properties and vegetation biomass on the 1st and 7th days (due to rainfall on the 8th day) after heavy rainfall (all *P* < 0.01; Fig. 5). Soil bulk density was negatively correlated with the CRSW (Fig. 5a,g) while total porosity (Fig. 5b,h) and field capacity (Fig. 5c,i) were positively correlated with the CRSW. Root (Fig. 5d,j), tree (Fig. 5e,k) and litter biomass (Fig. 5f,l) were significantly positively correlated with the CRSW. This result indicates that both soil and vegetation factors could influence the CRSW under different forest management practices.

Soil and vegetation factors were compared among the four stands (Tables 3 and 4). The tree biomass and litter biomass levels of the HIT stand were significantly lower than those of the other stands (*P* < 0.05; Table 4). The litter biomass in the NTN and LIT stands was significantly higher than that in the USR stand, which included twice as much litter biomass as the HIT stand (Table 3). Similarly, root biomass levels in the HIT stand were lower than those in the NTN stand except at the 0–20 and 60–80 cm depths (Table 4). In addition, the HIT stand presented significantly lower total porosity and field capacity levels and higher bulk density values than the NTN stand (Table 4). Soil properties in the LIT stand did not differ from those in the NTN stand (Table 4).

**Table 3:** Tree and litter biomass in *P. massoniana* stands under different forest management practices.

Variable	NTN	USR	LIT	HIT
Tree biomass(kg·m^-2^)	23.74 ± 4.50 a	19.20 ± 4.13 a	21.40 ± 2.83 a	6.48 ± 1.16 b
Litter biomass(g·m^-2^)	580.69 ± 32.54 a	432.60 ± 42.97 b	515.27 ± 62.48 a	170.96 ± 32.08 c

Means ± standard deviations (n = 3) are shown, and different letters shown in the same row denote significant differences between the different forest management systems at *P* < 0.05.

*NTN* no thinning stand, *USR* understory removal stand, *LIT* light-intensity thinning stand, *HIT* heavy-intensity thinning stand.

**Table 4:** Soil properties and root biomass in *P. massoniana* stands under different forest management practices.

Variable	Layers (cm)	NTN	USR	LIT	HIT
Bulk density (g·m^−3^)	0–20	1.24 ± 0.07 a	1.24 ± 0.05 a	1.23 ± 0.10 a	1.41 ± 0.07 b
	20–40	1.30 ± 0.05 a	1.31 ± 0.03 b	1.29 ± 0.03 a	1.48 ± 0.09 b
	40–60	1.30 ± 0.06 a	1.27 ± 0.09 a	1.23 ± 0.03 a	1.60 ± 0.03 b
	60–80	1.31 ± 0.06 a	1.39 ± 0.05 b	1.33 ± 0.01 ab	1.67 ± 0.03 c
	80–100	1.39 ± 0.03 a	1.45 ± 0.05 a	1.41 ± 0.03 a	1.66 ± 0.03 b
Total porosity (%)	0–20	56.98 ± 1.69 a	59.93 ± 6.96 a	59.43 ± 4.39 a	46.03 ± 2.30 b
	20–40	53.52 ± 2.15 a	51.96 ± 2.46 a	55.64 ± 5.29 a	44.11 ± 3.72 b
	40–60	51.60 ± 3.99 a	46.16 ± 9.23 ab	50.72 ± 1.52 a	38.68 ± 1.04 b
	60–80	50.48 ± 4.51 a	48.46 ± 5.09 a	52.86 ± 6.46 a	37.25 ± 2.48 b
	80–100	45.03 ± 1.72 a	46.31 ± 3.10 a	46.96 ± 3.61 a	38.05 ± 1.46 b
Field capacity (%)	0–20	46.05 ± 3.69 a	48.19 ± 5.95 a	48.54 ± 7.65 a	32.76 ± 2.95 b
	20–40	41.10 ± 0.98 a	39.73 ± 2.36 a	43.05 ± 3.87 a	30.08 ± 4.64 b
	40–60	39.96 ± 4.71 a	36.56 ± 9.35 a	41.12 ± 2.00 a	24.16 ± 0.92 b
	60–80	38.64 ± 4.87 a	34.89 ± 2.98 a	39.80 ± 4.93 a	22.37 ± 1.73 b
	80–100	32.41 ± 1.77 a	32.06 ± 1.50 a	33.32 ± 2.52 a	22.99 ± 0.78 b
Root biomass (g·m^−2^)	0–20	388.98 ± 44.41 a	283.04 ± 120.40 a	270.40 ± 56.82 a	242.58 ± 38.59 a
	20–40	304.49 ± 57.28 a	253.89 ± 35.27 ab	318.25 ± 107.86 a	167.29 ± 49.13 b
	40–60	245.65 ± 36.54 a	216.29 ± 57.30 ab	301.99 ± 93.43 a	126.87 ± 38.94 b
	60–80	187.75 ± 31.44 a	174.59 ± 83.78 a	243.32 ± 119.53 a	87.20 ± 14.34 a
	80–100	118.98 ± 27.54 a	120.50 ± 32.84 a	95.44 ± 12.49 a	50.85 ± 14.40 b

Means ± standard deviations (n = 3) are shown, and different letters shown in the same row denote significant differences between the different forest management systems at *P* < 0.05.

*NTN* no thinning stand, *USR* understory removal stand, *LIT* light-intensity thinning stand, *HIT* heavy-intensity thinning stand.

### The driver model for the CRSW after heavy rainfall

**Figure 6:**
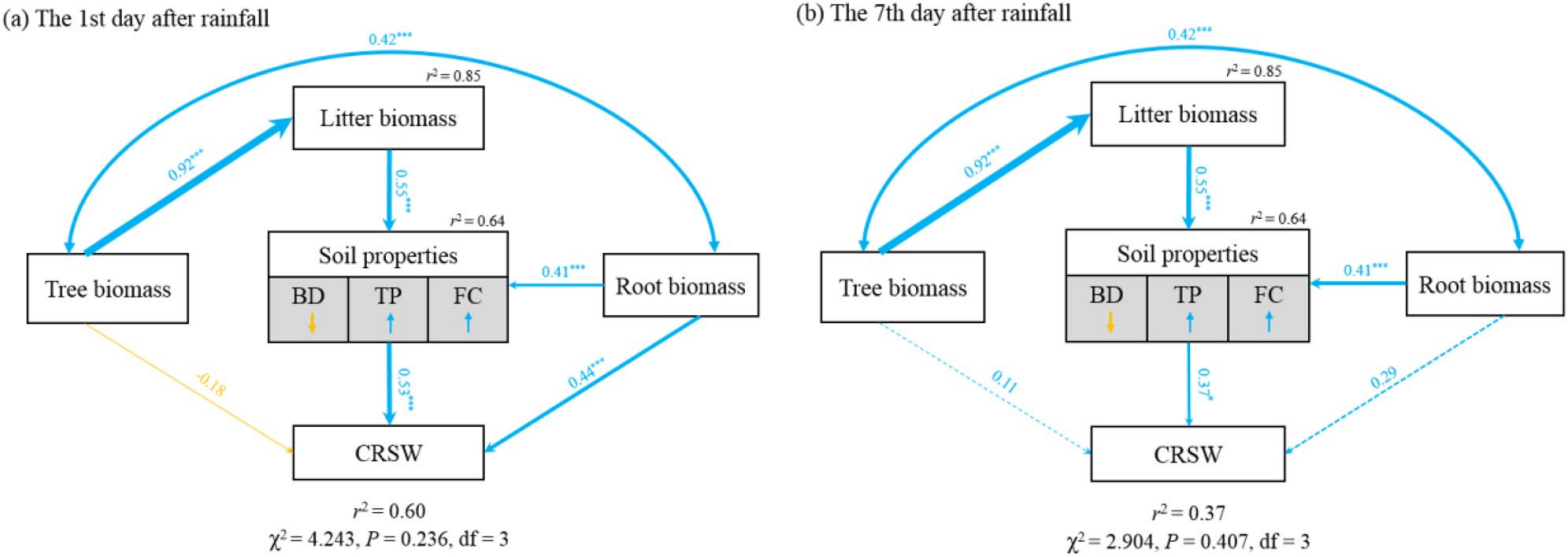
Structural equation model (SEM) showing the effects of soil properties, tree biomass root biomass and litter biomass on the CRSW (the contribution of heavy rainfall to soil water) on the first **(a)** and seventh **(b)** days after heavy rainfall (n = 60). Soil properties measured include BD (bulk density), TP (total porosity) and FC (field capacity). Arrows indicate effect directions and paths; red and blue lines represent positive and negative effects, respectively; line widths denote the strength of path coefficients. Standardized path coefficients shown on the lines represent the effect sizes of relationships. Goodness-of-fit statistics of the model are displayed below the model. **P < 0.01, ***P < 0.001.

**Figure 7:**
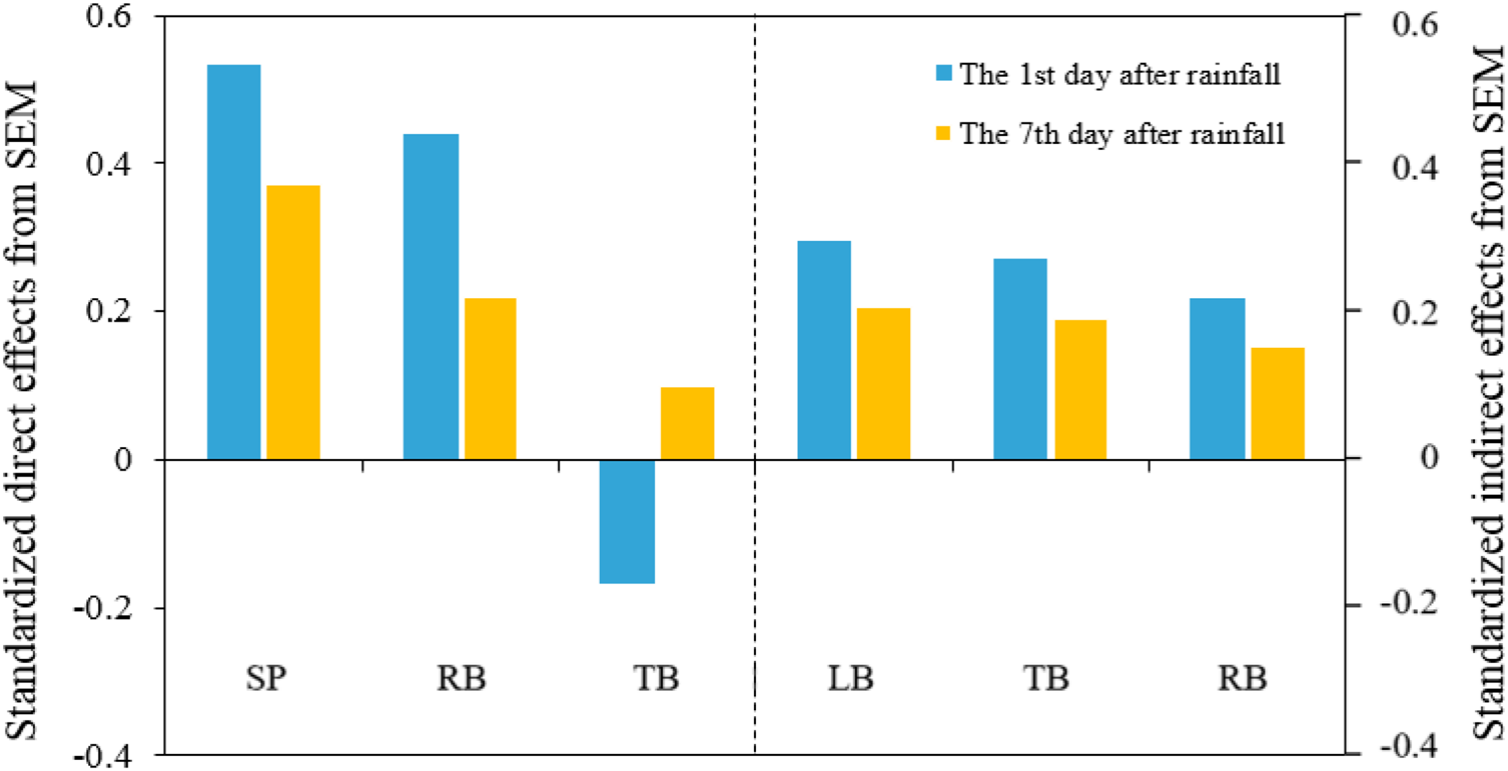
Standardized direct and indirect effects on the contribution of heavy rainfall to soil water from structural equation modelling (SEM). *SP* soil properties, *RB* root biomass, *TB* tree biomass, *LB* litter biomass.

To quantify the relative importance of different variables to the CRSW after heavy rainfall, two SEMs were constructed based on known relationships between the CRSW and key driving factors on the 1st and 7th days after heavy rainfall in the four stands. The SEM explained 60% and 37% of the variance in the CRSW on the 1st and 7th day after heavy rainfall, respectively (Figs. 6 and 7). Both soil properties and root biomass had a direct impact on the CRSW (Figs. 6 and 7). Litter biomass had an indirect impact on the CRSW by affecting soil properties (Figs. 6 and 7). In addition, tree biomass indirectly affected the CRSW through interactions with litter biomass and root biomass (Figs. 6 and 7). Compared with the 1st day after heavy rainfall, the soil properties and root biomass of the direct influencing factor standardized path coefficient decreased from 0.53 and 0.44 to 0.37 and 0.22 on the 7th day after heavy rainfall, respectively (Fig. 7). Meanwhile, the litter biomass and tree biomass of indirect influencing factors decreased from 0.30 to 0.21 and from 0.27 to 0.19, respectively (Fig. 7). Taken together, soil properties and litter biomass were the most important direct and indirect predictors affecting the CRSW, respectively (Figs. 6 and 7).

## Discussion

### SWC and soil water δD responses to rainfall events

We found the responses of SWC and soil water *δ*D to different rainfall events were varied. SWC and soil water *δ*D values were relatively stable after light rainfall, while heavy rainfall had a strong impact on SWC and soil water *δ*D in terms of changes in the CV (Table 2). These findings are in agreement with previous studies of other forest ecosystems^8,47^. It may be that light rainfall with short pulses wetted only the topsoil, while heavy rainfall could penetrate deeper soil layers. Meanwhile, the *δ*D in surface soil water was lower than that in deeper soil after three rainfall events (Fig. 3). This observation may be attributed to the influence of rainfall with negative *δ*D values, suggesting that precipitation heavily shapes the isotopic compositions of soil water^43,48,49^. In addition, we found the SWC and *δ*D of surface soil displayed larger variances than those of deep soil. This result is consistent with the observations of previous studies^28,47^, which may be due to the combined effects of precipitation and evaporation. Surface soil water receives more rainwater and undergoes more pronounced evaporation than deep soil^48^.

### Effects of forest management practices on soil water-holding capacity

The slightly higher CRSW found in the HIT stand than in the NTN stand in some soil layers after light and moderate rainfall may be attributed to differences in canopy density (Fig. 4). As Table 1 shown, the HIT stand with lower canopy density has more openings that can allow rainfall to infiltrate the soil^50,51^, while NTN, USR and LIT stands with higher canopy density have better capacities to intercept rainfall. In contrast, the CRSW in the HIT stand was significantly lower than that in the NTN and LIT stands after heavy rainfall (Fig. 4). This result indicates that soil in the NTN and LIT stands can better retain heavy rainfall and absorb more water when rainwater saturates the soil and exceeds the soil maximum water-holding capacity. These features lessen soil erosion, retard runoff generation and decrease the likelihood of flooding^52,53^. This may occur for the following two reasons. First, vegetation regulates soil water movement and storage^54,55^. The LIT and NTN stands with integrated vertical structures showed better soil hydrological functions than the HIT stand due to higher canopy coverage, litter biomass and root biomass (Tables 3 and 4). These factors improve the soil capacity of retaining rainfall and water conservation. Meanwhile, integrated structure forests have a stronger buffer effect on soil water^29^. A high buffer capacity may indicate rapid water absorption during rainfall and over long water conservation period^56,57^. Therefore, NTN and LIT stands have a greater capacity of retaining rainfall than HIT stand. Second, the difference of soil properties leads to vary of soil water-holding capacity. The HIT stand showed significantly lower levels of total porosity and field capacity and higher bulk density levels than the NTN and LIT stands (Table 4). Soil with higher total porosity and lower bulk density has a higher water-holding capacity^4,45^. When a large amount of rainfall reaching the soil can reach or even exceed the upper limit of soil water-holding capacity, NTN and LIT stands absorb more water and exhibit stronger water-holding capacity than HIT stand. Taken together, the NTN and LIT stand soils showed a greater capacity to retaining heavy rainfall than the HIT stand, and vegetation and soil factors can affect the contribution of heavy rainfall to soil water via increasing the soil water-holding capacity, respectively.

### Key factors regulating the soil water-holding capacity of *P. massoniana* plantations

The results of our SEM show that soil properties were the main factors affecting soil water-holding capacity under different forest management practices (Figs. 6 and 7). Similar results are presented in other studies^21,28,29^ and show that soil properties have strong effects on water storage dynamics, especially in humid areas. Among soil properties, field capacity was found to be the most closely correlated with the CRSW after heavy rainfall (Fig. 5c,i), which may be due to soil field capacity itself reflecting soil water-holding capacity under sufficient rainfall conditions^58^. In addition to field capacity, total porosity was another important indicator of soil water conservation that mainly affects soil water holding capacity by increasing the effective space for rainfall infiltration. Additionally, reductions in soil bulk density further improve some important soil properties, such as porosity, aeration, permeability and infiltration^45^. Taken together, these properties directly affect soil water-holding capacity and determine the threshold for water storage^59,60^.

In addition, no significant differences in the soil properties of 0–60 cm most layers of the control plots beside the four studied stands (Supplementary Table S6) suggest that the four stands had similar primitive soil characteristics prior to the application of forest management practices. This further demonstrate that forest management practices resulted in soil property changes rather than in primitive soil differences in the stand sites. Previous studies also indicate that low- and moderate-intensity thinning do not have significant effects on soil properties^12,61^. With increasing thinning intensity, soil bulk density gradually increases while total porosity and field capacity gradually decrease^33^. Further increasing thinning intensity easily exposes and compacts the soil, resulting in a decline in drainage and ventilation performance^45^. This is consistent with our finding of the HIT stand exhibiting significantly lower total porosity and field capacity and higher bulk density than the NTN stand. The changes in soil properties caused by heavy-intensity thinning may be due to the following two aspects. On the one hand, below-ground vegetation (root biomass) regulates the soil properties. Shen et al.^36^ in this area also showed that the decrease of soil aggregate content, which closely related to soil water-holding capacity, caused by heavy logging may be induced by the difference of plant roots among different treatments. On the other hand, above-ground vegetation regulates the soil properties by intercepting rainfall and organic matters inputting. The more exposed soils of the HIT site may have developed surface seals or crusts owing to the impact of rainfall (resulting from the reduced canopy and litter protection), which directly influenced soil water infiltration capacity. The less inputting of soil organic matter may further aggravate soil crust^38^. Besides, logging activities inevitably caused slight soil compaction that lead to lower total soil porosity and field capacity, and higher bulk density^33,45^, although the effect of human trampling on soil compaction was far less than that of heavy machinery and the effect of that may not be markedly.

Although the influence of root biomass on soil hydrological functions appear to be of little concern in many previous studies^62^, root biomass is also another key factor that regulates the CRSW after heavy rainfall in this study (Fig. 7). Plant roots have a positive effect on soil penetration^63^. The large pores formed by plant roots serve as important channels for water infiltration^64^. In addition, the root has the function of connecting soil particles, releasing secretions and promoting the formation of soil aggregates, which determine the permeability and corrosion resistance of soil^65^. Increased root biomass improves the indirect positive effects of soil physical properties on the CRSW after heavy rainfall^66^. Therefore, compared with that in the NTN stand, the significantly reduced root biomass in the HIT stand (Table 4) weakened the soil capacity of retaining rainfall.

In addition to being directly regulated by soil properties and root biomass, the CRSW after heavy rainfall was also indirectly regulated by litter biomass (Fig. 7). However, previous study found that the litter did not significantly affect the CRSW^4^. In our study, it may be that the NTN and LIT stands with more litter had less compacted soils, which improved soil water-holding capacity. Besides, increased litter biomass improves soil properties by increasing soil organic matter levels and microbial activity^67^. This causes soil total porosity to increase and bulk density to decrease^68^, ultimately resulting in an increase in soil water-holding capacity. Hence, higher litter biomass levels found in the NTN and LIT stands than in the HIT stand (Table 3) helped improve the soil water-holding capacity.

### Implications for forest management

Extreme precipitation events and seasonal drought have seriously affected the plantations of subtropical region, leading to potentially increasing the relative importance of soil controls on water^69^. The focus of water-oriented forest management has been put on increasing soil water and aquifer recharge rather than increasing runoff and streamflow^70^. Our study indicated that no and light-intensity thinning managements show higher soil water-holding capacity compared with the high-intensity thinning managements. This may be benefit to retarding the progression of runoff generation, mitigate soil erosion and decrease the possibility of triggering floods^42,53^. Therefore, the plantation under no and light-intensity thinning have stronger resistance and resilience to precipitation pattern changes than that under heavy-intensity thinning. In contrast, heavy-intensity thinning should be avoided. Although previous studies believe that thinning can alleviate the water stress of plants and increase soil water storage by biomass reduction in arid area^14,18^. However, given that the high-intensity thinning forest have limited dynamic storage in wetter periods, this will limit the effectiveness of vegetation management for mitigating the floods and soil erosion in subtropical region. Furthermore, in our study, soil water-holding capacity of most layers in the USR stand is not different from the other three stands, which indicating that understory removal may not significantly change the soil water-holding capacity in the short term. However, previous studies have shown that understory vegetation play an important role in regulating the soil water storage capacity^16,17^. Therefore, research on the effect of understory removal on the water-holding capacity of plantation soil should be carried out on a longer time scale. In addition, it is worth noting that the spatial heterogeneity of soil water may bring uncertainty to soil water research^71^. Therefore, additional studies specifically on the distribution of soil water sampling points are needed in order to better understand spatial variables and to define treatments for subsequent spatial distribution of water isotope sampling in forest ecosystems.

## Conclusion

In this study, we applied hydrogen isotopes to measure the effects of different forest management practices on the contribution of rainfall to soil water. The results show that under different forest management practices, plantation soil shows different water storage dynamics. Following light and moderate rainfall events, the soil in the HIT stand received slightly more rainwater than the NTN stand. However, following heavy rainfall, the contributions of rainfall to soil water in the NTN and LIT stands were significantly higher than those observed for the HIT stand. The contribution of rainfall to soil water of most layers in USR stand was not different from the NTN stand after three magnitudes of rainfall. Compared to the HIT stand, the LIT and NTN stands showed greater capacities to retain rainwater, resulting in a good buffering effect on rainwater. Soil properties are the main factors causing the difference in soil capacity of retaining rainfall. Therefore, under anticipated patterns of global change with more frequent and extreme precipitation events, heavy-intensity thinning to harvest timber should be avoided in *P. massoniana* plantations. Contrasting to heavy-intensity thinning, methods involving no and light-intensity thinning seem to be an effective way to show better soil and water conservation functions, which will be critical in retaining rainwater and moderating runoff and soil erosion.

## Supplementary Information

Supplementary Information.
