# Supplementary material for: Effects of thinning and understory removal on the soil water-holding capacity in Pinus massoniana plantations
Source: Sci Rep. 2021 Jun 22;11:13029. doi: 10.1038/s41598-021-92423-5 (PMC8219692; doi:10.1038/s41598-021-92423-5)
Supplement: Supplementary file 1 — Supplementary Information. [file 41598_2021_92423_MOESM1_ESM.docx]

Supplementary material for

Effects of thinning and understory removal on the soil water-holding capacity in *Pinus massoniana* plantations

Ting Wang^1^, Qing Xu^1*^, Deqiang Gao^1*^, Beibei Zhang^1^, Haijun Zuo^1^, Jing Jiang^2^

^1^Key Laboratory of Forest Ecology and Environment of National Forestry and Grassland Administration, Research Institute of Forest Ecology, Environment and Protection, Chinese Academy of Forestry, Beijing 100091, China

^2^University of Calgary, Calgary T2N1N4, Canada

**^*^Corresponding author:**

Qing Xu, tel: + 86 10-6288 9549, fax: + 86 10-6288 4972, Email: [xuqing@caf.ac.cn](mailto:xuqing@caf.ac.cn)

Deqiang Gao, tel: + 86 10- 6288 9510, fax: + 86 10-6288 4972, Email: [gaodeqiang@caf.ac.cn](mailto:gaodeqiang@caf.ac.cn)

**Contents of this file:**

Table S1 to S6

Figure S1 to S3

|  | NTN | USR | LIT | HIT |
| --- | --- | --- | --- | --- |
| >4000 μm (%) | 13.13±0.39 | 12.15±4.49 | 12.19±0.61 | 12.83±0.53 |
| 2000-4000 μm (%) | 18.19±5.23 | 15.56±2.06 | 13.56±2.47 | 18.43±6.47 |
| 250-2000 μm (%) | 51.74±5.13 | 46.78±3.05 | 53.87±4.78 | 50.83±8.21 |
| 53-250 μm (%) | 13.95±0.13 | 20.45±4.08 | 14.47±0.79 | 17.80±5.61 |
| <53 μm (%) | 2.99±0.37 | 5.06±1.07 | 5.91±1.24 | 5.27±0.81 |
| >250 μm (%) | 83.06±0.49 | 74.49±4.46 | 79.62±1.07 | 75.72±4.24 |

**Table S1** The weight proportion of soil aggregates in surface soil layer (0-10 cm) in the *Pinus massoniana* stands with different tending thinning practices. NTN: no thinning stand; USR: understory removal stand; LIT: light-intensity thinning stand; HIT: heavy-intensity thinning stand^1^.

|  | NTN | USR | LIT | HIT |
| --- | --- | --- | --- | --- |
| PH | 5.85 ± 0.02 | 6.02 ± 0.05 | 6.17 ± 0.02 | 5.97 ± 0.05 |
| Soil organic carbon (g∙kg^-1^) | 22.29±3.34 | 19.41±4.29 | 21.75±6.18 | 21.62±4.72 |
| Total nitrogen (g∙kg^-1^) | 2.12±0.26 | 1.80±0.29 | 1.90±0.38 | 1.92±0.37 |
| Total phosphorous (g∙kg^-1^) | 0.21±0.02 | 0.21±0.03 | 0.22±0.03 | 0.23±0.03 |
| Total potassium (g∙kg^-1^) | 14.55±1.33 | 15.06±2.05 | 13.04±2.85 | 12.9±1.69 |
| Carbon nitrogen ratio | 10.48±0.75 | 10.72±0.99 | 11.35±1.18 | 11.28±1.11 |

**Table S2** Soil physical and chemical properties in surface soil layer (0-10 cm) in the *Pinus massoniana* stands with different tending thinning practices^2,3^. NTN: no thinning stand; USR: understory removal stand; LIT: light-intensity thinning stand; HIT: heavy-intensity thinning stand.

| Variable | PC 1 |
| --- | --- |
| Bulk density | -0.940** |
| Total porosity | 0.964** |
| Field capacity | 0.996** |

**Table S3** Correlation coefficient between soil properties and the first principal component. ***P* < 0.01

| Rainfall events | Layers (cm) | NTN | | USR | | LIT | | HIT | |
| --- | --- | --- | --- | --- | --- | --- | --- | --- | --- |
|  |  | df | *P*-value | df | *P*-value | df | *P*-value | df | *P*-value |
| Light rainfall | 0-20 | 4 | 0.002 | 4 | 0.002 | 4 | 0.002 | 4 | 0.002 |
|  | 20-40 | 4 | ＜0.001 | 4 | ＜0.001 | 4 | 0.003 | 4 | 0.001 |
|  | 40-60 | 4 | 0.071 | 4 | 0.673 | 4 | 0.073 | 4 | 0.024 |
|  | 60-80 | 4 | 0.917 | 4 | 0.918 | 4 | 0.621 | 4 | 0.422 |
|  | 80-100 | 4 | 0.120 | 4 | 0.906 | 4 | 0.217 | 4 | 0.438 |
| Moderate rainfall | 0-20 | 4 | 0.002 | 4 | 0.013 | 4 | 0.007 | 4 | 0.001 |
|  | 20-40 | 4 | 0.040 | 4 | 0.001 | 4 | 0.002 | 4 | 0.002 |
|  | 40-60 | 4 | 0.024 | 4 | 0.004 | 4 | 0.005 | 4 | 0.003 |
|  | 60-80 | 4 | 0.004 | 4 | 0.016 | 4 | 0.003 | 4 | 0.007 |
|  | 80-100 | 4 | 0.123 | 4 | 0.049 | 4 | 0.004 | 4 | 0.002 |
| Heavy rainfall | 0-20 | 4 | 0.001 | 4 | ＜0.001 | 4 | ＜0.001 | 4 | ＜0.001 |
|  | 20-40 | 4 | 0.001 | 4 | 0.006 | 4 | ＜0.001 | 4 | 0.018 |
|  | 40-60 | 4 | 0.003 | 4 | 0.003 | 4 | ＜0.001 | 4 | 0.039 |
|  | 60-80 | 4 | 0.005 | 4 | 0.003 | 4 | ＜0.001 | 4 | 0.003 |
|  | 80-100 | 4 | 0.001 | 4 | 0.003 | 4 | 0.001 | 4 | 0.008 |

**Table S4** Summary of independent t-test in testing pre-rainfall and first day after rainfall of soil water *δ*D among the different forest management practices. NTN: no thinning stand; USR: understory removal stand; LIT: light-intensity thinning stand; HIT: heavy-intensity thinning stand.

| Rainfall events | Layers (cm) | df | F-value | *P*-value |
| --- | --- | --- | --- | --- |
| Light rainfall  (8.9 mm) | 0-20 | 3 | 1.104 | 0.355 |
|  | 20-40 | 3 | 0.521 | 0.669 |
|  | 40-60 | 3 | 4.466 | 0.007 |
|  | 60-80 | 3 | 0.902 | 0.446 |
|  | 80-100 | 3 | 0.017 | 0.997 |
| Moderate rainfall  (13.3 mm) | 0-20 | 3 | 0.704 | 0.554 |
|  | 20-40 | 3 | 0.624 | 0.602 |
|  | 40-60 | 3 | 1.678 | 0.182 |
|  | 60-80 | 3 | 1.537 | 0.215 |
|  | 80-100 | 3 | 1.433 | 0.243 |
| Heavy rainfall  (67.7 mm) | 0-20 | 3 | 2.480 | 0.073 |
|  | 20-40 | 3 | 2.911 | 0.045 |
|  | 40-60 | 3 | 4.429 | 0.008 |
|  | 60-80 | 3 | 3.210 | 0.032 |
|  | 80-100 | 3 | 4.405 | 0.009 |

**Table S5** Results of a one-way ANOVA testing the contribution of rainfall to soil water in the *P. massoniana* plantation under the different forest management practices.

| Variable | Layers (cm) | NTN | USR | LIT | HIT |
| --- | --- | --- | --- | --- | --- |
| Bulk density  (g·m^-3^) | 0-20 | 1.31±0.04 a | 1.26±0.03 a | 1.24±0.02 a | 1.28±0.04 a |
|  | 20-40 | 1.39±0.05 a | 1.30±0.07 b | 1.25±0.00 b | 1.39±0.03 a |
|  | 40-60 | 1.39±0.03 a | 1.36±0.03 a | 1.35±0.06 a | 1.48±0.10 a |
|  | 60-80 | 1.45±0.04 a | 1.39±0.03 a | 1.39±0.06 a | 1.57±0.02 b |
|  | 80-100 | 1.46±0.08 ab | 1.39±0.09 a | 1.42±0.02 a | 1.55±0.02 b |
| Total porosity  (%) | 0-20 | 61.29±4.21 a | 53.68±4.65 a | 53.78±6.19 a | 58.50±5.86 a |
|  | 20-40 | 56.68±3.80 a | 51.49±2.89 a | 50.22±5.00 a | 48.63±7.58 a |
|  | 40-60 | 54.60±5.20 a | 47.89±4.21 a | 49.33±5.63 a | 50.45±8.46 a |
|  | 60-80 | 47.98±2.41 a | 47.02±4.67 a | 47.25±3.42 a | 40.75±1.65 a |
|  | 80-100 | 48.08±1.08 a | 48.68±5.38 a | 43.82±6.23 a | 38.52±3.25 a |
| Field capacity  (%) | 0-20 | 46.88±4.70 a | 41.64±1.97 a | 43.30±4.66 a | 46.81±6.38 a |
|  | 20-40 | 40.68±2.47 a | 40.20±4.10 a | 40.19±4.05 a | 34.84±3.73 a |
|  | 40-60 | 39.34±3.71 a | 35.27±3.09 a | 36.80±5.57 a | 34.37±8.22 a |
|  | 60-80 | 33.02±1.97 a | 33.90±3.91 a | 34.14±3.14 a | 25.99±1.37 b |
|  | 80-100 | 32.92±1.12 a | 35.36±5.74 a | 30.78±4.63 ab | 24.76±1.87 b |

**Table S6** Soil properties in four *P. massoniana* stands before different forest management practices. Means ± standard deviations (n = 3) are shown and different letters in the same row refer to significant difference among four stands at *P* < 0.05. NTN: no thinning stand; USR: understory removal stand; LIT: light-intensity thinning stand; HIT: heavy-intensity thinning stand.


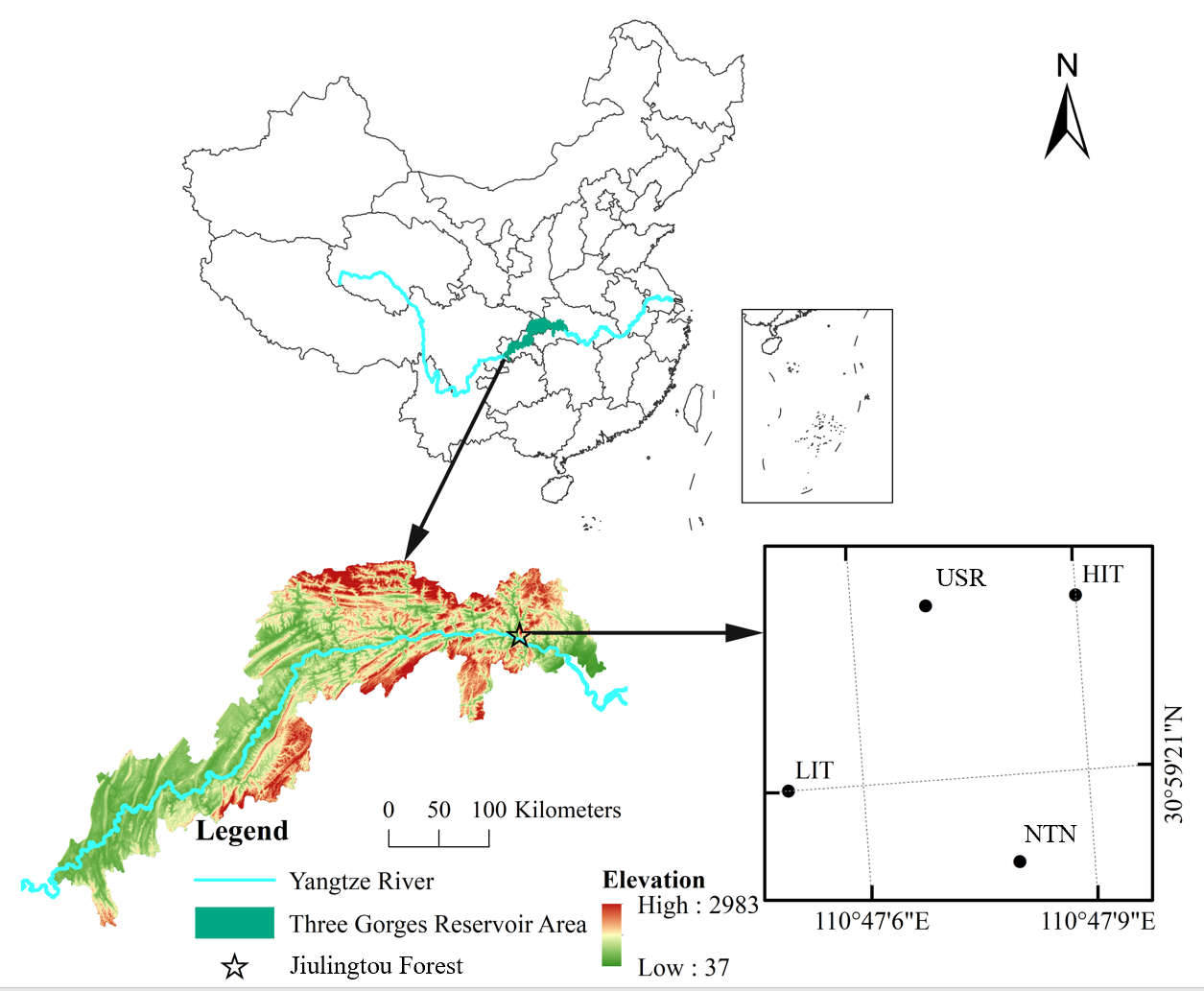


**Fig. S1.** Location of the study area, including NTN, USR, LIT, and HIT stands of *P. massonian*a in Jiulingtou Forest of the Three Gorges Reservoir Area in China. The maps were created using ArcGIS 10.2 software (https://www.esri.com/en-us/arcgis/products/arcgis-desktop/overview).


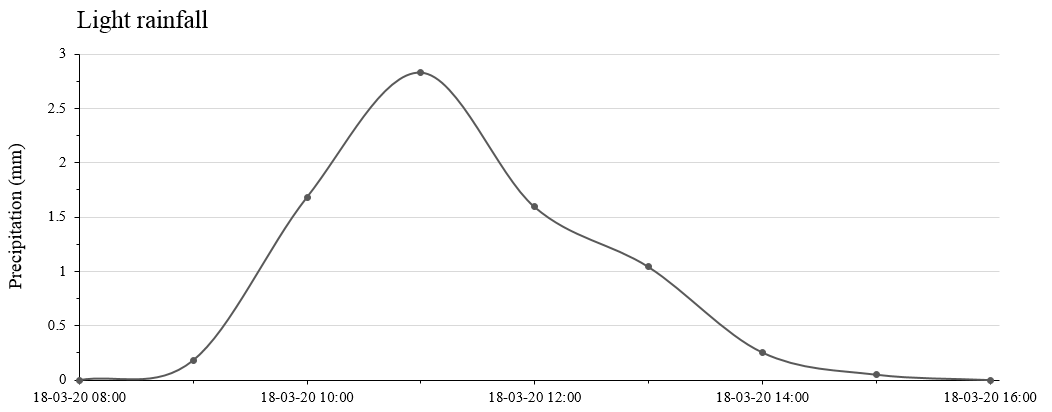

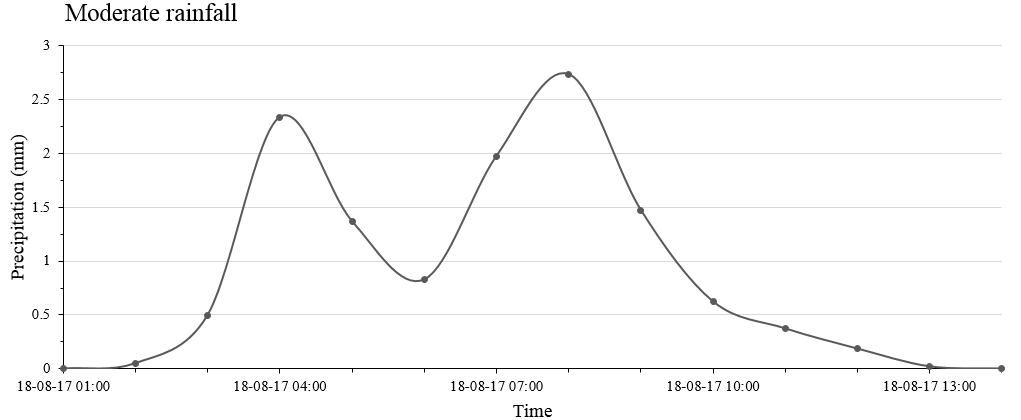

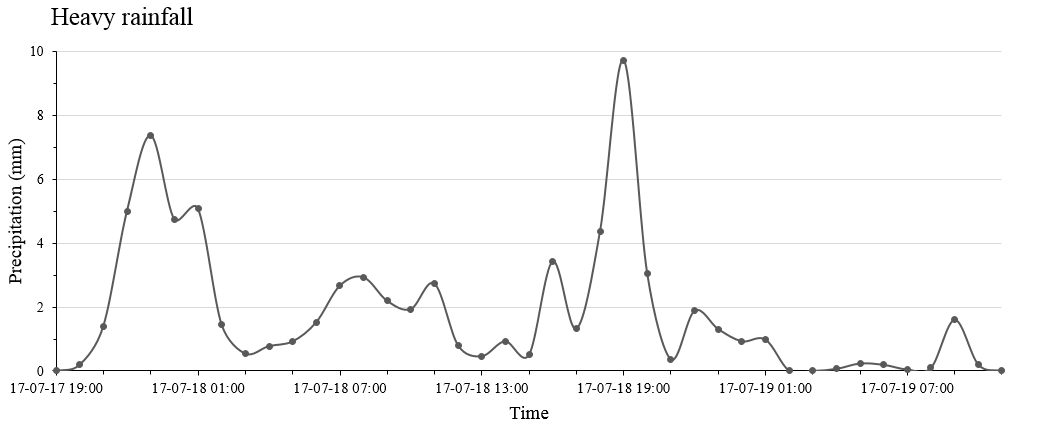


**Fig. S2** The characteristics of light, moderate and heavy rainfall events.


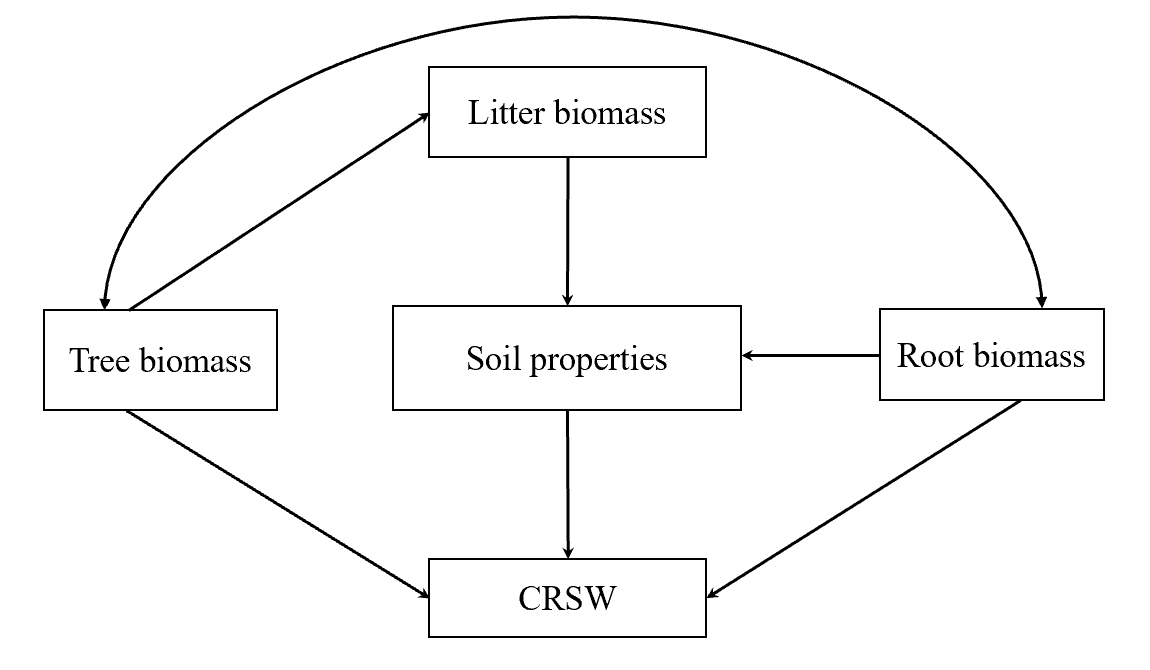


**Fig. S3.** Structural equation model (SEM) including vegetation biomass and soil properties. CRSW: the contribution of heavy rainfall to soil water.

References

1. Shen, Y. F. Effects of understory vegetation removal and thinning on soil organic carbon and the related processes in *Pinus Massoniana* plantations. PhD dissertation. China, Beijing: Chinese academy of forestry. pp: 32-43 (2018).
2. Shen, Y. F. *et al.* Labile organic carbon pools and enzyme activities of *Pinus massoniana* plantation soil as affected by understory vegetation removal and thinning. *Sci. Rep.* **8**, 573 (2018).
3. Wang, X. R. *et al*. Short-terms effects of tending thinning on soil labile organic carbon in *Pinus massoniana* stands. *Chin. J. Ecol.* **40**, 1049-1061 (2021).
